# Qualitative study of Ebola screening at ports of entry to the UK

**DOI:** 10.1136/bmjgh-2018-000788

**Published:** 2018-06-27

**Authors:** Joanna May Kesten, Suzanne Audrey, Maya Holding, Caroline Coope, Nick Young, Colin S Brown, Jenny Harries, Matthew Hickman, Isabel Oliver

**Affiliations:** 1 NIHR Health Protection Research Unit in Evaluation of Interventions, University of Bristol, Bristol, UK; 2 The National Institute for Health Research Collaboration for Leadership in Applied Health Research and Care West (NIHR CLAHRC West), University Hospitals Bristol NHS Foundation Trust, Bristol, UK; 3 Population Health Sciences, Bristol Medical School, University of Bristol, Bristol, UK; 4 Field Epidemiology Service, National Infection Service, Public Health England, Bristol, UK; 5 NIHR Health Protection Research Unit in Emerging and Zoonotic Infections, Institute of Infection and Global Health, University of Liverpool, Liverpool, UK; 6 Public Health England South West Centre, Public Health England, Bristol, UK; 7 National Infection Service, Public Health England, Bristol, UK; 8 Public Health England South of England Regional Office, Public Health England, London, UK

**Keywords:** qualitative study, public health, screening, infections, diseases, disorders, injuries, health systems evaluation

## Abstract

**Introduction:**

In response to the 2013–2016 West African outbreak of the Ebola virus disease (EVD), Public Health England introduced enhanced screening at major UK ports of entry. Our aim was to explore screeners’ and screened travellers’ perceptions of screening as part of an evaluation of the screening programme.

**Methods:**

We undertook qualitative focus groups and semistructured interviews with screeners and travellers who had returned from affected countries before and after the introduction of screening in England. The study was conducted in two airports: one international rail terminal and one military airport. Research topic guides explored perceptions of the purpose and implementation of the process, potential improvements and reactions to screening. The data were analysed using the framework method.

**Results:**

Twenty-four screeners participated in 4 focus groups (one for each port of entry) and 23 travellers participated in interviews. Three themes are presented: ‘Context’, ‘Screeners’ experience of the programme’ and ‘Screening purpose and experiences’. The programme was implemented rapidly, refined over time and adapted to individual ports. Screeners reported diverse experiences of screening including negative impacts on their normal roles, difficult interactions with passengers and pressure to identify positive EVD cases. Screening was considered unlikely to identify individuals with symptoms of EVD, and some participants suggested it was driven by political concerns rather than empirical evidence. The screening process was valued for its provision of information and reassurance.

**Conclusion:**

This qualitative study found that the UK EVD screening process was perceived to be acceptable to assess individual risk and provide information and advice to travellers. Future programmes should have clear objectives and streamlined processes to minimise disruption, tailored to the nature of the threat and developed with the needs of humanitarian workers as well as general travellers in mind.

**Key questions**

**What is already known?**

- Public Health England introduced enhanced screening to assess the health status of persons returning from Ebola virus disease-affected countries and ensure they were aware of what actions to take if subsequently taken unwell.

**What are the new findings?**

- Differential views were elicited between the intended purpose of screening, the experience of those receiving screening and the outcomes achieved.
- The screening programme was viewed as acceptable to screeners and screened travellers; however, the experience and perceived needs of healthcare workers taking part in the humanitarian response can differ from that of general travellers.

**What do the new findings imply?**

- Future programmes should have clear objectives from the start and processes streamlined to minimise disruption, tailored to the nature of the threat and, where safe, developed with stakeholder experience in mind.
- Future programmes should consider the specific needs of healthcare workers taking part in the humanitarian response as well as general travellers.

## Introduction

The 2013–2016 West African outbreak of Ebola virus disease (EVD) was the largest since the discovery of the virus in 1976,^1^ with more cases and deaths than all other outbreaks combined.^2^ UK healthcare and military volunteers supported the response primarily in Sierra Leone, with Guinea and Liberia also significantly affected.

The West African EVD outbreak was declared a public health emergency of international concern by the WHO on 8 August 2014. Some direct flights to the UK from the region had been discontinued by airline operators before this date: all were discontinued on 27 August 2014. Public Health England (PHE) introduced enhanced screening for EVD on 14 October 2014 at major ports of entry in England as part of activities in response to the risk to public health. Prior to this date, people returning to England from EVD affected countries were not routinely identified on entry to the UK. Travellers were advised to seek medical attention as soon as possible if they developed any symptoms compatible with EVD via information provided in the media, PHE’s website, notices at ports of entry and travel companies. Advice from occupational health teams before departure and varied systems for supervision on return were also provided by some organisations (eg, non-governmental organisations). Enhanced screening was introduced to: provide information to persons returning from EVD affected countries, assess their health status and ensure they were aware of what actions to take if subsequently taken unwell.^3 4^ Although the detection of large numbers of EVD cases was not anticipated, screening was introduced both to advise travellers and to provide a degree of reassurance for the UK public.^5^

Entry screening involved completion of a questionnaire assessing health and travel history, potential risk of EVD exposure via occupation and contact with infected individuals,^6^ assignment of a risk category (0=low to 3=high) (table 1) and tympanic temperature measurement. Screeners provided advice to all travellers including actions to take if symptoms developed. Category 0 travellers were provided with reassurance and advice to continue with their routine activities. Category 1 travellers were additionally advised to take their temperature if they felt unwell and to phone the National Health Service (NHS) telephone helpline if it was ≥37.5°C. If EVD signs or symptoms were observed or disclosed, travellers were assessed by a clinical screener who determined the need for referral to specialist care.^6^ Category 2 travellers were provided with a monitoring kit and asked to record their temperature twice daily during the 21-day period since the last day in an EVD-affected country (used as a proxy measure for last exposure to risk) and report if they felt unwell or had a temperature of ≥37.5°C. Category 3 travellers were also provided with a monitoring kit and asked to record their temperature twice daily, additionally having to report their temperature to PHE by midday.^7^

**Table 1:** Category of risk description

Category of risk	Description	Public Health England recommended actions
Category 0	Individuals who have not visited an EVD affected area and have had no contact with EVD or an EVD infected individual.	Reassure and provide written and verbal information. Continue normal activities.
Category 1	Individuals who have visited an EVD affected area but had no direct contact with an EVD infected individual or been exposed to any other high-risk event. This category also included those working in laboratories assured to be operating to a UK standard.	Reassure and provide written and verbal information. Advise to take temperature if feeling unwell and phone 111 if ≥37.5°C. Continue normal activities.
Category 2 (low-risk exposure)	Individuals having direct (close) contact with an infected person or their body fluids but who did not have direct physical contact during clinical care and had no known breaches of protective equipment/clothing during this contact.	Reassure and provide written and verbal information. For 21 days following last exposure: Self-monitor and record temperature and symptoms twice daily and report if feeling unwell and/or temperature≥37.5°C. Travel – no restrictions. Normal activities except: Postpone non-essential medical or dental treatment (including vaccination), and inform healthcare provider of contact if any essential treatment needed. If reluctant to comply with recommendations, consider actions on a case-by-case basis.
Category 3 (high-risk exposure)	High-risk individuals who had direct contact with symptomatic infected individuals and potential exposure to bodily fluids including breaches to protective equipment/clothing or had worked in a laboratory not assured to be working to UK standard.	Reassure and provide written and verbal information. For 21 days following last exposure: Self-monitor and record temperature and symptoms twice per day and report to designated person by noon daily. If there is inability to take temperature a face-to-face arrangement will be made with relevant health services. Continue normal activities (while no symptoms) except: Local travel only. If a healthcare worker, no patient contact for 21 days. Postpone non-essential medical or dental treatment (including vaccination) and inform healthcare provider of contact if any essential treatment needed. Do not share toothbrushes or razors. Use barrier contraception or avoid unprotected sex for 21 days. If reluctant to comply with recommendations, consider actions on a case-by-case basis.

Adapted from public health guidance ‘Ebola: public health recommendations for asymptomatic contacts’ https://www.gov.uk/government/publications/ebola-public-health-recommendations-for-asymptomatic-contacts.

EVD, Ebola virus disease.

Two sources of information were used to identify eligible travellers: the Returning Healthcare Workers (HCWs) Scheme (RHWS) and Advanced Passenger Information (API). The RHWS involved organisations registering with PHE and providing details of deployed individuals’ return dates. API includes the passengers’ full name, date of birth, gender, nationality and passport number and is collected by airlines prior to travel either when a flight is booked or automatically through passport details obtained at check-in. API enabled the pre-entry identification of most eligible travellers expected on each flight. Self-identification was relied on to detect eligible travellers not identified via RHWS or API. As none of these systems were 100% effective, it was assumed that not all eligible travellers would be identified.

It is important to understand how screening programmes are experienced by those delivering and receiving them to ensure that the design and implementation of similar initiatives in the future are acceptable and adhered to. The aim of this paper is to explore screeners’ and screened travellers’ perceptions of this EVD screening programme.

## Methods

### Research design

JMK undertook qualitative focus groups (May–August 2015) with screeners implementing screening processes (eg, taking temperatures, administering questionnaires and undertaking clinical assessments). Focus groups are useful for conducting exploratory investigations into emerging areas of interest and provide a relatively efficient data collection method. Interactions between participants generate new information as participants build on the responses of others and focus groups facilitate insights into the extent to which the experience of screeners agree or diverge.

JMK conducted semistructured one-to-one interviews (August–November 2015) with travellers who were referred for further investigation of febrile illness after returning to the UK from affected countries prior to the introduction of screening and screened travellers (hereafter referred to as prescreening and postscreening). CC conducted one interview. As travellers’ places of residence were geographically widespread, one-to-one interviews were conducted by telephone. Additionally, the experiences of passengers were expected to be more diverse than screeners (who were following a protocol); therefore, one-to-one interviews were appropriate.

The evaluation was conducted at two airports (ports 1 and 3), one international rail terminal (port 2) and one military airfield (port 4), chosen to reflect different port size, screened traveller volume and mode of travel. Port 2 could not use API and relied on travellers being asked, and reporting, whether they had been to an affected country. One focus group per port was conducted with screeners from the same port to enable participants to recall collective experiences.

### Participant recruitment and consent

Invitations and information sheets were posted or emailed to potential participants. To achieve a sample with maximum variation in views we used a purposeful sampling approach. Local screening managers organised focus groups with screeners and ensured diversity in relation to: experience of screening travellers within each category of risk, professional backgrounds and role within the process (eg, clinical and non-clinical roles) and gender. PHE and military databases of screened travellers were used to sample participants based on: port of entry, gender, category of risk and development of EVD signs and symptoms either at, or post, screening. Travellers who had returned from affected countries prior to the introduction of screening were selected using the same criteria from the PHE Health Protection (HP) Zone database. A sample framework was devised based on the stated variables, and travellers were then selected at random using the ‘sample’ command in Stata V.13.1. Travellers who declined to participate or did not respond after a maximum of three invitations were replaced by someone randomly sampled with similar characteristics. In total, 73 travellers were invited to interview (14 prescreening and 44 postscreening by the research team, and 15 directly by the military).

Informed consent was obtained from all participants.

### Data collection

The interview topic guide used with travellers who returned prescreening explored whether they felt informed about actions to take if they felt unwell. Topic guides for screeners and screened travellers similarly allowed comparison and exploration of the perceptions of the purpose and implementation of the screening process, barriers and facilitators to following the protocol (screeners only), potential improvements and reactions to screening (appendix A and B). Travellers were also asked about the impact on their knowledge, willingness to adhere to advice and behaviour. All interviews and focus groups were digitally recorded. Data collection and analysis occurred concurrently to allow for consideration of the adequacy of the sample size. The decision to end data collection was informed by factors relating to the concept of ‘information power’: the breadth of the aim, the sample specificity (characteristics of the participants relating to the phenomenon under study), quality and depth of the interview data and the analysis approach which, in this evaluation, did not aim to capture the entire range of experiences but present sufficient information to explore perceptions of screening.^8^ Additionally, pragmatic considerations of resources including time and availability of screeners guided the decision.

### Analysis

Audio recordings were fully transcribed and anonymised. Data were analysed using the framework method,^9^ supported by QSR NVivo V.10 software. After data familiarisation, codes identifying key issues within the data were assigned to three transcripts. Initial codes were discussed by JMK and SA and refined to produce a coding framework, which was applied to the remaining transcripts and revised in response to new information. Coded data were inserted into a matrix in NVivo, which plotted the codes against each participant, condensing the volume of data with a summary capturing the meaning.

## Results

### Participants

Table 2 details the characteristics of 24 screeners who participated in four focus groups. A recruitment rate cannot be presented for screeners as focus groups were organised by local screening managers and in ports 1 and 2 depended on who was available on the day. No one declined to participate directly to the researcher. The average length of focus groups was 81 min (range: 53–103 min). Twenty-three (31.5% recruitment rate) travellers participated in interviews (mean 36 min, range: 20–57 min) (table 3). Two travellers declined to participate citing the length of the interview and being too busy as reasons; the remaining 51 either did not respond to the invitation or the email failed to deliver. Of the 15 invited directly by the military, no reason for non-participation was received by the research team and no screeners contacted the research team to decline participation.

**Table 2:** Focus group screener participant characteristics

Characteristic	Port 1	Port 2	Port 3	Port 4	Total
**Gender**					
Male	1	2	1	1	5
Female	7	5	4	3	19
**Screening role** *					
Shift manager	1	1	–	–	2
Operational lead	–	1	1	2	4
Overseeing role	–	–	1	1	2
Non-clinical screener	–	–	1	1	2
Clinical: screener/senior lead	4	4	–	–	8
Non-clinical support	3	1	3	–	7
Governance/health and safety role	–	–	1	1	2
Overall total	8	7	5	4	24

*Some screeners held more than one role.

**Table 3:** Ebola screening evaluation traveller participant characteristics

Participant characteristic	N
Gender	
Male	14
Female	9
Occupation/employer	
Healthcare provider (non-military)	3
Military medical	4
Military non-medical	3
Journalists (eg, correspondent, producer and camera person)	4
Retired	1
Other non-medical (eg, engineer and heavy plant fitter)	2
Non-medical response to Ebola outbreak (eg, epidemiologist, humanitarian worker and nutritionist)	6
**Returned to UK prescreening (before 14 October) as well as postscreening**	11
Number of times screened on entry to UK	
0 (returned prescreening only)	1
1	13
2 or more	9
Port of entry (note: some travellers had used more than one port of entry)	
Port 1	19 (including 5 military travellers)
Port 2	3 (including 1 military travellers)
Port 3	4
Port 4	2 (both military travellers)
Port 5 (not originally a sampled port but travellers had been screened here)	2
Category of risk	
No category given as only returned prescreening	1
1	15
2	1
3	2
Could not remember	4
Signs and symptoms present at either port of entry or during 21-day incubation period	
Yes	10
No	13
Referred to secondary care	
No	16
Yes – directly from port	2
Yes – during 21-day incubation period	5
Total	23

Three themes with illustrative quotes are presented below to reflect the range of views from participants.

### Context

Screening was established in a short timescale under challenging circumstances and the process was refined over time.

You have to remember it is an evolving thing. So initially we were all in a bit of a situation I think but over a period things started improving in all spheres actually. Port 1, screener 2

Although the screening protocol was standardised, implementation was dependent on the port context. Ports varied in relation to facilities, identification processes, disruption to the traveller journey caused by the process (ie, distance between facilities and border control/luggage collection), duration of the process and volume of screened travellers.

### Screeners experience of the programme

Ensuring adequate staffing levels was initially challenging, in part due to the unpredictable volume of travellers. Over time, the proportion of PHE staff seconded to the programme teams became more robust. Screeners in ports 1 and 2 reported variable degrees of original line managerial support for screening involvement.

In the beginning it was a lot of different staff day in and day out and I think now in the past couple of months I think a lot of us have come on secondment (…) You’re here all the time so you know the process. So when you get people who haven’t been for a couple of months you can say ‘Everything’s the same’ or ‘We do this now’. Port 1, screener 4

Given the diverse background of screeners, the relevance of training content was variable. In two ports, screeners felt that there was a lack of clarity on the risks to screeners including what screeners should do to protect themselves if they were exposed to a suspected EVD case.

Despite recognising procedural modifications as improvements, the dynamic nature of the programme made keeping up to date with the procedures challenging.

One of the most stressful things was all the paperwork actually and you know thinking am I catching up, have I read everything, am I update on the different versions? Port 2, screener 2

Negative aspects of screening related to: the impact on the screeners’ routinely employed roles; difficult interactions with passengers; perceived pressure concerning the identification of positive EVD cases; and autonomy to adapt the process. In contrast, collaborating with internal (eg, different PHE departments) and external bodies (airport personnel including Border Force) was a positive aspect of delivering the programme.

### Screening purpose and experiences

Comparison of the formally documented objectives of the screening process to the perceptions of those who experienced it can help to understand the delivery of, and response to, the screening process.

#### Protection or politically driven?

Screeners and most travellers described screening as intending to protect the public and prevent the spread of EVD in the UK.

I do think there was a genuine desire to keep people safe but also psychologically I think it’s important that you know, we are seen to be doing something. Traveller 7, prescreening and postscreening, ports 1 and 2

However, screeners viewed the introduction of screening as driven by political concerns rather than empirical evidence. A small number of travellers did not believe that screening could protect the UK population. Instead, screening was viewed as ‘*over cautious’* (traveller 14, male, prescreening and postscreening, port 1), ‘*window dressing*’, an ‘*umbrella hoisting exercise*’ (traveller 6, male, prescreening and postscreening, port 1) and politically driven.

It was just a political move, it wasn’t backed by health professionals. Port 1, screener 4

#### Categorising risk of EVD exposure

Screening was described by all screener groups and some travellers as categorising travellers in relation to their level of risk, based on self-reported behaviours performed while in an affected country.

[Travellers] don’t have a good idea of what their risk is. They know that they have done this or done that but they don’t know how much that activity puts them at risk. So we are an expert who assess their activity and say ‘You are at a high risk or low risk or very low risk’ and give them appropriate advice which I’m sure many people wanted. Port 1, screener 2

The screening questionnaire was described by screeners and travellers as in-depth and appropriate but was also criticised as limited by its reliance on self-reporting. Journalists commented that the questions appeared to be designed for HCWs and were unable to capture some of their behaviours such as observing burials rather than funerals.

The questions are very basic and it would be very easy for you if you did not want to be held up or put in quarantine to just tick ‘no’ to everything, ‘no I’ve not been to a funeral, no I’ve definitely not got Ebola’ and as long as your temperature wasn’t raised at that point, which you could lower by taking Ibuprofen if you so wished, or you may not be in that stage of incubation, you could quite easily pass through the screening. Traveller 3, prescreening and postscreening, ports 1 and 2

Travellers generally perceived risk categorisation as useful, and discussing the questionnaire responses with a screener was considered valuable when the risk category was unclear or if travellers wished to be recategorised. Screeners were also able to help travellers who found questionnaire completion challenging due to extreme tiredness.

I said ‘well you know, the way I’ve answered these questions, yes I suppose I would be lowest [category of risk] but I think you should probably bump me up the scale simply because you know, I have been into the cemeteries as they’re burying Ebola victims, you know, I was sitting there filming, I’ve been you know fairly close to people who are likely to have had Ebola being collected from the streets of Sierra Leone, you know, I’ve been into treatment centres – although I haven’t actually gone to the very centre of them, we have had cameras that have gone in which I’ve then had contact with (…) I think I should probably be a bit higher up’ and I think in the end they said ‘oh alright, why not’ and then I think I was a 2. Traveller 17, postscreening, port 1

However, there was a general perception by screeners that risk categories were less useful for HCWs who had prior knowledge of EVD.

#### Identifying possible EVD signs and symptoms

Most travellers and screeners in port 3 felt that screening aimed to identify individuals displaying EVD signs and symptoms.

There is the actual ‘let’s see if we can’t catch people who are infected before they go off and infect people’. Traveller 7, prescreening and postscreening, ports 1 and 2

However, several travellers thought identifying individuals with symptoms was unlikely.

In terms of the risk and incubation period and stuff like that, in terms of actually being able to travel while you’re sick, like the chances of someone actually displaying symptoms at screening is small. Traveller 22, prescreening and postscreening, ports 1, 2 and 3

Furthermore, port 2 screeners and one traveller felt the process should not be called ‘screening’, because it was unable to confirm the presence or absence of EVD. Indeed, using temperature measurements to screen was not seen as evidence based by some travellers and a small number of travellers raised issues relating to the accuracy of the thermometer readings due to the type of thermometer and the ‘non-touch’ method whereby screeners placed the thermometer into the ear without making skin-to-skin contact with the traveller. However, the latter is recognised as a valid method of taking temperature.

I think screening is not the right word because it’s about getting people into the system rather than actually checking whether people have got Ebola. Port 2, screener 7

#### Referral to NHS if signs and symptoms identified

Screeners and travellers described screening as aiming to ensure appropriate specialist assessment for those displaying signs and symptoms.

To ensure that if a person is identified who’s at…. who’s symptomatic, then….systems were to be set that that person could be safely transported to the [name of hospital]. Port 3, screener 1

However, delayed transfers to secondary care were reported by travellers and screeners, and a small number of travellers disagreed with transfer decisions. Screeners were mostly considered by travellers as calm and sympathetic when dealing with travellers with signs and symptoms, but one traveller described a screener as panicking.

He took my temperature and it shot right up, it was pretty high by then, and he… you could see that he basically shat himself!! Ha-ha… and then I was taken away into a smaller room, and then a doctor came in – she asked me loads of questions, took my temperature again but she had no protection on, then she disappeared for 20 min, and then came back, and said I could go – which to my mind was the wrong thing to do, I would have thought I should have been carted off to hospital there and then. Traveller 16, prescreening and postscreening, ports 1 and 5

#### Raising awareness of appropriate actions and advice provision

Screeners and travellers identified the aim of screening as raising awareness of the appropriate actions to take should EVD signs and symptoms develop including using the NHS telephone helpline rather than going directly to hospital. Although several travellers talked about screening providing such practical advice, most did not feel they gained new knowledge and the evidence base for advice to restrict travel for asymptomatic individuals was questioned.

We had like leaflets that…it was all quite basic stuff like about the Ebola outbreak so I guess that most people probably knew more than the PHE guys about it if they’d been living and working it. Traveller 22, prescreening and postscreening, ports 1–3

We appeared to have science at the start of this which suggested that unless you were leaking body fluids, you were no danger to anybody, and yet here we are speaking to somebody who’s just come back from Sierra Leone whose chance even as a red zone worker of contracting the disease was vanishingly low, that you now cannot travel on public transport in case you infect anybody. Traveller 6, prescreening and postscreening, port 1

#### Reassurance?

All screener groups, and some travellers, described screening as being implemented to reassure travellers and the public. Although screeners recognised the low risk posed to the public, addressing risk perceptions and providing reassurance was viewed as an important function.

It’s a public confidence thing, and so I guess that the purpose is to protect us and the purpose is to be seen to be protecting us. Traveller 7, prescreening and postscreening, ports 1 and 2

However, port 2 screeners identified a mismatch between the public’s perception and the reality of what screening could achieve. This gave rise to concerns that if a positive case was not detected the public would think screening had ‘failed’.

There seems to be a bit of a gap between the public reassurances because the public think that we’re checking whether or not anyone’s got Ebola. I suppose actually we are just putting people on a system and following them up. So then if somebody gets Ebola then people think we’ve failed. Port 2, screener 7

A small number of HCWs and non-HCWs described reassurance from screening as personal or for family, friends, colleagues or the wider public.

That [letter] PHE sent, saying ‘you are Category 1, you are not seen as a risk, you can continue your life as normal, your normal duties’ – that was a massive help, because there was so much fear here at that time and I remember coming back and people being quite worried about being close to me! So having that piece of paper was, I almost kind of had it stamped to my forehead! To say that ‘look I’ve been screened, it’s fine.’ Traveller 9, prescreening and postscreening, ports 1 and 2

In contrast, one traveller asserted that he and his family relied on his own awareness, rather than the screening, for reassurance.

Screeners and a small number of travellers perceived some other travellers to be anxious about screening. Foreign nationals, particularly West Africans, were perceived to find the process more intimidating than British nationals due to the uncertainty about the process, the stigma of EVD and wariness about whether screening related to immigration processes. Segregating travellers for screening was thought by some screeners and travellers to give the impression that they had been detained by immigration, and a few travellers found the identification process and handling by Border Force officials stigmatising.

We know what we’re going to do but they don’t know what we are going to do, so they are a bit anxious. Port 1, screener 2

#### Inconvenience?

Despite the intention to reassure, screening reportedly led to some annoyance among travellers. All screener groups described travellers as wanting to get through the process quickly and displaying annoyance at delays. Almost half the travellers described annoyance about the delay to their journey and suggested that similar processes in the future should avoid this by ensuring sufficient staffing levels; however, the other half viewed the delay as acceptable and screening as efficient.

The whole thing for me ran smoothly it was only a 5–10 min process, you know, it didn’t delay me. Traveller 11, postscreening, port 3

Screeners in all groups provided examples of negative responses to multiple screening occasions, caused by the discontinuation of direct flights, for example, screening on both exit and entry for a single journey and on stopovers, and multiple screening during the 21-day incubation period.

Participant 4: They were [name of country] diplomats who’d been screened here 2 days ago and had travelled to [European city] on business and had come back into Port 1. And because our procedure that we have been passed down from above … is that they have to be rescreened….

Participant 3: I’d be fed up at that.

Participant 4: And (…) I’m not clinical staff, you know, I struggle to understand it from the beginning but I’ve asked clinical staff who work on that front line basis with those travellers who don’t agree with it either. Port 1, focus group

#### Monitoring system and adherence to advice

Screening involved monitoring the temperature of category 3 travellers throughout the potential incubation period, enabling early isolation and treatment if any travellers developed symptoms. The appropriateness of this process was questioned by travellers because they were already motivated to report signs and symptoms. Indeed, some screeners felt that HCWs should be allowed to take responsibility for protecting themselves and the public. Despite this, travellers reported adhering to screeners’ advice – monitoring their symptoms and adhering to PHE recommended actions appropriate to their category of risk (eg, not taking public transport). However, a small number of travellers described being unsure when to report signs and symptoms and some viewed screening and related restrictions on return to the UK as a poor way to treat HCWs.

[The monitoring process is] intrusive and, (…) paternalistic and patronising because, (…) as a HCW, I’m not going to be so cavalier about my own health and the health of my family or the nation that if I suddenly started to feel ill, having been in contact with an Ebola patient, I wouldn’t have thought, ‘Ooh, perhaps I’d better talk to somebody’. So the fact that we then sort of are made to take our temperatures and are made to ring somebody up to say that we’re feeling well, to be perfectly honest, I really think that was a complete waste of time. Traveller 6, prescreening and postscreening, port 1

#### Acceptability of screening

Most travellers felt that screening was acceptable and, several travellers commented that familiarity with temperature checking while in West Africa, contributed to the acceptability of UK screening. Despite some negative responses, most screened travellers were also described by screeners as accepting and valuing the process. Screeners suggested that returning HCWs were broadly supportive of screening even though it was not expected to have any direct impact on them, although those with higher risk exposures were described by screeners as being less receptive to screening than those at lowest risk.

A huge vast majority of people have been really receptive and have really welcomed. (…) So I think from a public health safety perspective it’s been very, very valued erm, not only from people in the UK but from people coming in as well. Port 1, screener 3

## Discussion

This paper presents an exploration of screeners and travellers perceptions of the UK EVD port of entry screening programme. The findings highlight ways to enhance the acceptability of similar initiatives in the future. Screeners and travellers felt that the screening process served diverse functions consistent with the stated objectives of the programme, although there were some tensions around its intended purpose and actual perceived benefits. Screening was seen as providing public health guidance and advice, which was generally appreciated by travellers and was considered by both travellers and screening staff as reassuring for the public. However, its effectiveness as a ‘screening’ service to identify undiagnosed EVD cases was questioned, consistent with national scientific debate^10^ and technical guidance^11^ at the time. There was a degree of frustration arising from the inconvenience caused.

### Screening process and improvements

We and others^12^ highlight the responsive and evolving nature of screening, due in part to the short timescale prior to implementation and lessons learnt, for example, from instances of inadequate staffing. If a similar process is to be considered for other public health emergencies, then formal plans should be developed taking into account the lessons from this outbreak. It is also important to build in flexibility that can respond to the availability of appropriate facilities, the nature of the threat^13^ and factors that may affect acceptance and perception of risk.^14 15^

We highlight accounts of frustration caused by multiple screening episodes in one journey. Previous research suggests that detection of symptomatic travellers through exit screening—requiring international collaboration—may be a beneficial approach. Nonetheless, there are limitations in this approach, notably the risk of passengers becoming symptomatic during travel, challenges in monitoring passengers through diverse routes of travel and individual countries duty to protect their citizens. Furthermore, experiences of delayed transfers to specialist care highlight a need to develop and exercise plans for the management of high-consequence infectious diseases including the prompt and safe transfer of symptomatic persons from ports.

Screening was dependent on the ability to identify eligible travellers; the systems that support this have limitations and ways to facilitate and improve the identification of travellers need consideration. Direct flights from the main EVD-affected countries to the UK were withdrawn resulting in people travelling via alternative routes. On arrival to the UK, additional efforts were therefore required to identify travellers coming from affected countries, but there was the potential for travellers to be missed.^10 16^ Greater publicised clarity about the programme’s objectives may further encourage travellers to self-identify. Screening relied on self-reported information from travellers who may not have reported the presence of signs and symptoms for a number of reasons including a perception of journey delays.^10^

Screener experiences of delivering the programme suggest organisational support for internally seconded staff involvement is important. Training for similar initiatives, if screening is part of the response, should be tailored to the experience of attendees, and training for managing challenging passenger encounters may be necessary.

#### Acceptance

This evaluation highlights interesting contradictions between programme acceptance and the perceived purpose and experience of screening. The process of screening appeared to be valued overall and was perceived to be primarily for the provision of information and reassurance. Some participants considered screening was unlikely to identify individuals with EVD signs and symptoms, suggesting it was driven by political concerns rather than empirical evidence. Indeed, the appropriateness of the term ‘screening’ to describe the process was queried. Research suggests that if exit screening was effective, then screening on entry should only identify those travellers who developed symptoms during the flight.^10 16^ Modelling estimated that exit screening would identify 35.6% of infected travellers, screening after a further 24-hour period would identify 5.9% of EVD cases, and after a 12-hour period, 3.4% of EVD cases.^17^ In line with estimates for other infections,^18^ the positive predictive value of screening for EVD was expected to be very low.^16^ Due to the non-specific symptoms of EVD, and the evolving symptom progression, screening was expected to produce false-positive and false-negative results.^12^

Acceptance in spite of these limitations, and a degree of inconvenience and disruption^19^ involved, may be explained by the sense of reassurance experienced from screening. An alternative explanation is that screening promoted a false sense of reassurance. Nevertheless, our findings suggest that having a screening process was appreciated. A study exploring experiences of an EVD screening programme in the USA found that screened US citizens were less concerned about becoming unwell and the onward transmission of EVD than citizens of EVD-affected countries.^20^ Additional benefits of screening could include the identification of persons suffering emotional distress who could be referred to mental health services.^14^

#### Acceptable to whom?

Acceptance of screening by travellers has previously been reported.^9^ In this current evaluation, screening was generally seen as less relevant and impactful for returning HCWs. This contrasts with members of the public or non-healthcare professionals who appeared to value the use of risk categories. It is noteworthy that screening was perceived to have been introduced in part to reassure the public.

There is some evidence that HCWs may underestimate their risk of contracting EVD, and some may overestimate their knowledge of the symptoms of disease.^21^ The requirement for category 3 HCWs to report temperature readings to PHE was viewed by some as unacceptable and unnecessary but may have allowed for more objective assessment of the likelihood of disease and provide an opportunity to discuss EVD signs and symptoms. A previous evaluation suggested that a majority of those monitored do not trust their thermometer readings to be accurate.^20^

#### Enhancing acceptability

There have been previous reports of stigma associated with return from an EVD-affected country,^14 15^ and monitored persons have reported negative consequences including not being allowed to work and being shunned by family.^20^ Screening could potentially provide reassurance and formal permission to continue routine activities. To enhance acceptability and prevent stigmatisation, the objectives and intended outcomes of screening should be clear to staff and travellers, particularly returning HCWs. In this instance, this could have included emphasising that screening was designed to provide tailored advice and information in addition to appropriate access to further investigations when needed. A description of the programme and increased awareness of the credentials of screeners may have also helped improve acceptability. Involving those likely to be affected by screening—screeners and travellers—in all aspects of the design and implementation of future initiatives is also likely to enhance acceptability. Furthermore, acceptability is likely to increase through efforts to limit delays to travellers, and approaches to develop a more streamlined system for frequent travellers should be considered.

#### Strengths and limitations

This is the first qualitative evaluation of enhanced UK EVD screening, which elicited a diverse range of experiences from those delivering and receiving screening. The researcher (JMK) conducting most interviews was not a PHE employee and was initially unfamiliar with the programme, both of which were explained to the participants at the beginning of the interview to help ensure participant honesty. This also facilitated a critical distance in the interpretation of the data. The researcher gained familiarity with the research setting and programme by observing screening and or the screening set-up in all four ports. Focus groups with screeners began approximately 7 months after the start of screening, which meant the screeners had time to adjust to the process.

This study cannot make conclusive statements about whether screening achieved its objectives; it focused on eliciting accounts from those involved as opposed to more objective measures of programme activity. Although a purposeful sample of screeners was requested, a limitation of this approach was the inability to control this process and thereby assess whether a biased sample in relation to the perceptions of participants was achieved. Furthermore, the screeners may have felt conflicted between offering a professional versus personal opinion especially given that the focus groups were conducted at the screening site or within the screeners working environment. An additional useful aspect would be to capture the feelings of stakeholders involved in the higher level management of the programme, who may have given greater insights into programme design and implementation decision making processes, though they contributed to the design of the study and the drafting of the paper. It is possible that the travellers who were willing to participate may have held stronger views on screening than those who refused, or vice versa. Participants may have also felt reluctant to provide honest accounts to the researcher due to concerns about confidentiality and the researcher’s level of independence from PHE, despite this being stressed at the beginning of the interview. The recruitment rate for travellers (31.5%) is relatively low but not unexpected given the approach method relied on written invitation only and the time elapsed since screening, and only three participants were assigned risk category 2 or 3. Therefore, the participants’ views may not reflect all those screened. Traveller experiences may be subject to recall bias given a variable amount of time since screening and reports of extreme tiredness during screening. However, as the findings demonstrate a range of positive and negative experiences and patterns of commonalities as well as divergent views, these biases are expected to be minimal.

## Conclusions

According to travellers and screening staff, the UK EVD screening programme was acceptable and was perceived to be effective at assessing individual risk and providing information and advice to travellers according to that risk. In future, if similar programmes are being considered, it is important that there is clarity as to the objectives of screening and efforts are made to streamline processes and minimise disruption. Any future screening programme should be tailored to the nature of the threat posed, be developed with close involvement of key recipients and consider the specific needs of healthcare workers taking part in the humanitarian response as well as general travellers.
